# Adeno-associated viral vectors engineered for macrolide-adjustable transgene expression In mammalian cells and mice

**DOI:** 10.1186/1472-6750-7-75

**Published:** 2007-11-06

**Authors:** David A Fluri, Marie Daoud-El Baba, Martin Fussenegger

**Affiliations:** 1 Institute for Chemical and Bioengineering, ETH Zurich, HCI F115, Wolfgang-Pauli-Strasse 10, CH-8093 Zurich, Switzerland; 2 Institut Universitaire de Technologie, IUTA, Département Génie Biologique, 43 Boulevard du 11 Novembre 1918, F-69622 Villeurbanne Cedex, France

## Abstract

**Background:**

Adjustable gene expression is crucial in a number of applications such as de- or transdifferentiation of cell phenotypes, tissue engineering, various production processes as well as gene-therapy initiatives. Viral vectors, based on the Adeno-Associated Virus (AAV) type 2, have emerged as one of the most promising types of vectors for therapeutic applications due to excellent transduction efficiencies of a broad variety of dividing and mitotically inert cell types and due to their unique safety features.

**Results:**

We designed recombinant adeno-associated virus (rAAV) vectors for the regulated expression of transgenes in different configurations. We integrated the macrolide-responsive E.REX systems (E_ON _and E_OFF_) into rAAV backbones and investigated the delivery and expression of intracellular as well as secreted transgenes for binary set-ups and for self- and auto-regulated one-vector configurations. Extensive quantitative analysis of an array of vectors revealed a high level of adjustability as well as tight transgene regulation with low levels of leaky expression, both crucial for therapeutical applications. We tested the performance of the different vectors in selected biotechnologically and therapeutically relevant cell types (CHO-K1, HT-1080, NHDF, MCF-7). Moreover, we investigated key characteristics of the systems, such as reversibility and adjustability to the regulating agent, to determine promising candidates for *in vivo *studies. To validate the functionality of delivery and regulation we performed *in vivo *studies by injecting particles, coding for compact self-regulated expression units, into mice and adjusting transgene expression.

**Conclusion:**

Capitalizing on established safety features and a track record of high transduction efficiencies of mammalian cells, adeno- associated virus type 2 were successfully engineered to provide new powerful tools for macrolide-adjustable transgene expression in mammalian cells as well as in mice.

## Background

An array of different viral transduction systems are being used currently in pre-clinical and clinical trials [1-3]. Among these, vectors based on the replication-defective adeno-associated virus type 2 have attracted special attention as tools for clinical gene transfer. Different characteristics, such as (i) the ability to transduce dividing as well as non-dividing cells, (ii) high transduction rates in a wide range of tissues, and notably, (iii) the unique safety properties, make AAVs a promising vector in gene therapy initiatives [4-10].

Over the past few years, extensive studies have been carried out on different systems to regulate transgenes with small-molecule stimuli, preferentially clinically licensed agents. Started by the tetraycline-responsive TET system [11,12], numerous other control modalities have followed including those responsive to streptogramin [13], macrolide [14] and aminocoumarines [15], immunosuppressive agents (rapamycin) [16], hormones [17-19], or susceptible to temperature [20,21], quorum sensing molecules [22,23] and gaseous acetaldehyde [24]. To date, most of the experimental work with AAV vectors in the gene-therapy field has focused on (i) the expression of therapeutic transgenes driven by strong constitutive promoters [25-27], (ii) the regulated expression of transgenes based on the tetracycline-responsive TET_ON _and TET_OFF _systems [28-31] and, to lesser extent, (iii) the regulated expression of transgenes by rapamycin-controlled transgene expression [32,33].

Until recently, *in-vivo *studies using recombinant AAV particles have been limited by the production of high-titer and helper virus-free preparations. However, with the development of helper-free production methods [34,35] and improved purification and concentration protocols [36,37], high-titer production of AAV particles in the absence of helper-virus contamination was achieved, thereby opening the way to *in vivo *and clinical studies with AAV-derived particles.

We report the design and validation of different AAV type 2-based expression vectors, which enable macrolide-controlled transgene expression capitalizing on the recently developed erythromycin-responsive expression technology (E.REX) [14]. E.REX systems exist in two different configurations: (i) the E_OFF _arrangement consisting of a macrolide-dependent transactivator (ET, a fusion of the *Escherichia coli *MphR(A) repressor protein [E] and the *Herpes simplex *virus VP16 transactivation domain) which binds and activates chimeric promoters (P_ETR_) assembled by placing ET-specific operator modules 5' of a minimal eukaryotic promoter in a macrolide-responsive manner. Since the presence of erythromycin turns transgene expression off by abolishing the ET-P_ETR _interaction these control modalities are known as OFF-type or E_OFF _systems [14]. (ii) The E_ON _technology consists of a macrolide-dependent transrepressor (E-KRAB (ET4), [14]), a fusion of the *E. coli *MphR(A) repressor protein [E] and the KRAB (Krueppel-associated box) transsilencing domain of the human *kox-1 *gene), which binds and represses chimeric promoters (P_ETR_ON) assembled by placing E-KRAB-specific operator modules 3' of a constitutive eukaryotic promoter in a macrolide-inducible manner. Since the presence of erythromycin turns transgene expression ON by releasing E-KRAB from P_ETR_ON, these control arrangements are known as ON-type or E_ON _systems [14].

We have designed a set of recombinant AAV vectors harboring E_OFF _or E_ON_-controlled expression units (i) on two independent vectors (binary system), (ii) on a single vector expressing the transgene and the transactivator in a dicistronic or bidirectional configuration and (iii) on a single vector containing a constitutive promoter, driving transactivator expression, and the regulatable P_ETR _promoter, driving the gene of interest. We have optimized performance, delivery and regulation, analyzed key characteristics such as reversibility and adjustability of the systems and validated the results with quantitative *in-vitro *as well as mouse studies.

## Results

### Design of recombinant adeno-associated viral particles for transduction of E_OFF_-controlled transgene expression

**Figure 1:**
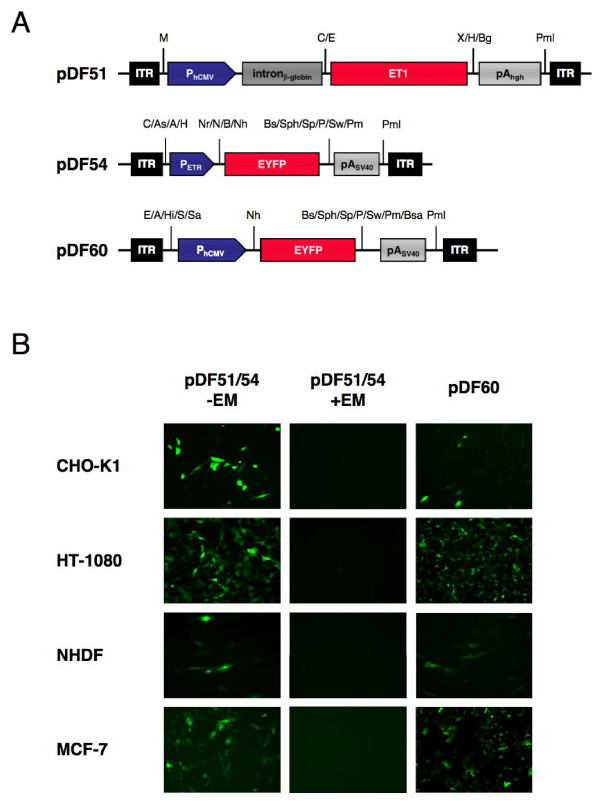
AAV type 2-based macrolide-responsive EYFP expression. (A) Schematic representation of AAV type 2-based vectors encoding ET1 under control of the constitutive P_hCMV _promoter (pDF51) and EYFP driven by P_ETR _(pDF54) or a constitutive hCMV promoter (pDF60). (B) Fluorescence micrographs of CHO-K1, HT-1080, NHDF and MCF-7 either co-transduced with pDF51/pDF54 and cultivated in the presence (+) and absence (-) of erythromycin (EM) or transduced with pDF60 (1000 genomic particles/cell for each vector). Abbreviations: EM, erythromycin; ET1, erythromycin-dependent transactivator; EYFP, enhanced yellow fluorescent protein; ITR, inverted terminal repeat; pA_hgh_, human growth hormone polyadenylation signal; pA_SV40_, simian virus 40 polyadenylation signal; P_ETR_, erythromycin-responsive promoter; P_hCMV_, human immediate early cytomegalovirus promoter; Selected restriction sites: A, *Acc*I; As, *Asc*I; Bg, *Bgl*II; Bs, *Bst*BI; Bsa, *Bsa*BI; C, *Cla*I; E, *Eco*RI; H, *Hind*III; Hi, *Hinc*II; M, *Mlu*I; N, *Nde*I; Nh, *Nhe*I; Nr, *Nru*I; P, *Pac*I; Pm, *Pme*I; Pml, *Pml*I; S, *Sal*I; Sa, *Sac*II Sp, *Spe*I; Sph, *Sph*I; Sw, *Swa*I; X, *Xba*I.

We have engineered serotype 2-based adeno-associated viral particles for transduction of macrolide-responsive expression of the enhanced yellow fluorescent protein (EYFP). The generic design consisted of a set of two vectors: pDF51 (ITR-P_hCMV_-intron_β-globin_-ET1-pA_HGH_-ITR) harboring an ITR (inverted terminal repeats)-flanked P_hCMV_- (human cytomegalovirus immediate early promoter) driven and pA_hgh_- (human growth hormone-derived polyadenylation site) terminated ET1 (macrolide-dependent transactivator) expression unit containing a β-globin intron (intron_β-globin_) reported to increase transcript processing and overall ET1 production levels and pDF54 (ITR-P_ETR_-EYFP-pA_SV40_-ITR) containing an ITR- flanked expression unit encoding P_ETR_- (macrolide-responsive promoter) driven and pA_SV40_- (simian virus 40-derived polyadenylation site) terminated EYFP (enhanced yellow fluorescent protein-encoding gene) expression cassette (Figure 1A). Transgenic AAV-derived particles produced by transient co-transfection of either pDF60, pDF51 or pDF54 with the helper construct pDG providing constitutive processing and assembly functions (adenovirus E2A, E4 and VA as well as AAV rep and cap genes; [38]) *in trans *into HEK293-T were validated for regulated EYFP expression by co-transduction of pDF51- and pDF54-derived AAV particles into representative cell types such as Chinese hamster ovary cells (CHO-K1), human fibrosarcoma cells (HT-1080), primary normal human dermal fibroblasts (NHDF) and human breast cancer cells (MCF-7) cultivated in the presence (+EM) or absence (-EM) of the macrolide antibiotic erythromycin (EM). Fluorescence micrographs of transduced populations maintained for 48 h in erythromycin-free medium revealed bright green fluorescence in all cell types. Transduced populations cultivated for two days in the presence of 1 μg/ml erythromycin showed no important EYFP expression. As a positive control all cell types were transduced with AAV particles harboring EYFP driven by a constitutive human cytomegolovirus immediate early promoter (P_hCMV_) (Figure 1B).

### One vector-based macrolide-responsive AAV expression vectors

**Figure 2:**
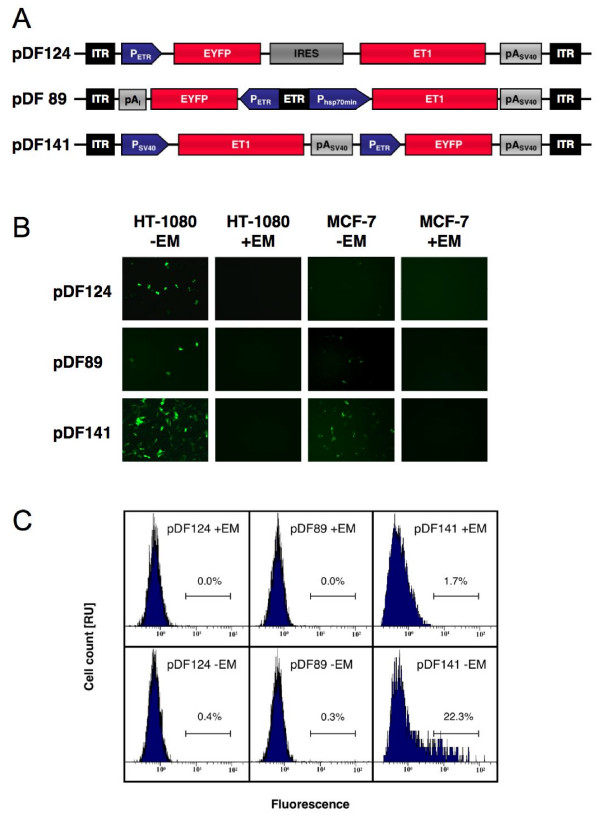
Self- and auto-regulated AAV type 2-based expression of fluorescent proteins. (A) Schematic representation of an auto-regulated (pDF124), a bidirectional (pDF89) and a self-regulated (pDF141) AAV type 2-based expression unit. (B) Fluorescence micrographs of human fibrosarcoma cells (HT-1080) and a human breast cancer cell line (MCF-7) transduced with pDF124-, pDF89- and pDF141-derived AAV particles (2000 genomic particles/cell) cultivated in the presence (+) and absence (-) of EM. (C) FACS-mediated quantification of EYFP in HT-1080 transduced with equal amounts of viral particles (2000 genomic particles/cell) and cultivated in the presence (+EM) and absence (-EM) of erythromycin. Abbreviations: EM, erythromycin; ET1, erythromycin-dependent transactivator; ETR, ET1-specific operator; EYFP, enhanced yellow fluorescent protein; IRES, internal ribosome entry site; ITR, inverted terminal repeat; pA_I_, synthetic polyadenylation signal; pA_SV40_, simian virus 40 polyadenylation signal; P_ETR_, erythromycin-responsive promoter; P_HSP70min_, minimal version of the *Drosophila melanogaster *heat-shock protein 70 promoter; P_SV40_, simian virus 40 promoter.

In order to enable delivery of macrolide-responsive transgene expression in a most compact format and a single transduction event we engineered expression of the macrolide-dependent transactivator ET1 and the desired target gene into a single AAV vector configuration. The pioneering one-vector design concept consisted of tandem constitutive ET1 (P_SV40_-ET1-pA_SV40_) and macrolide-responsive EYFP expression units (P_ETR_-EYFP-pA_SV40_) placed between two ITRs (Figure 2A). pDF141- (ITR-P_SV40_-ET1-pA_SV40_-P_ETR_-EYFP-pA_SV40_-ITR) derived AAV particles efficiently transduced HT-1080 and MCF-7 and mediated high-level EYFP expression when transgenic populations were grown for 48 h in the absence of erythromycin (Figure 2B). To prevent any deregulation of P_ETR _by promoters or enhancers encoded *in cis *and to minimize the overall size of the transgene unit, we assembled (i) pDF124 (ITR-P_ETR_-EYFP-IRES_EMCV_-ET1-pA_SV40_-ITR) a dicistronic autoregulated P_ETR_-driven AAV expression configuration harboring an ITR-flanked P_ETR_-driven dicistronic expression cassette with EYFP in the first and ET1 in the second cistron separated by a internal ribosome entry site of encephalomyocarditisviral origin (IRES_EMCV_) and (ii) pDF89 (ITR-pA_I_-EYFP←P_hCMVmin_-ETR-P_HSP70min_→ET1-pA_SV40_-ITR) containing a bidirectional expression unit consisting of a central ET1-specific operator ETR flanked by P_ETR _driving EYFP expression in one direction and a P_HSP70min _(minimal version of the *Drosophila melanogaster *heat-shock protein 70 promoter) driving ET1 in the opposite direction.

Following transduction of pDF124-derived AAV particles, active P_ETR _produces a single transcript from which EYFP is translated in a classic cap-dependent manner whereas ET1 production depends on cap-independent IRES_EMCV_-mediated translation initiation. Undetectable P_ETR_-mediated transcripts result in few initial ET1 proteins, which, in the absence of erythromycin trigger auto-regulated high-level expression of this transactivator along with the cocistronically encoded EYFP. In the presence of erythromycin, ET1 originating from basal P_ETR _activity is inactivated, which interrupts the auto-regulated feed-forward transcription and results in repression of EYFP production. After transduction of pDF89-derived AAV particles, leaky ET1 transcript initiate an auto-regulated expression circuit resulting in simultaneous expression of ET1 and EYFP until ET1-ETR binding is abolished by erythromycin. All three configurations yielded AAV particles, which displayed good regulation performance upon trandsduction of HT-1080 and MCF-7 and cultivation in the absence or in the presence of erythromycin (Figure 2B). While using equal genomic particle numbers the transduction efficiency varied between pDF124, pDF89 and pDF141. Trandsuction rates of the bi-directional (pDF89) and the dicistronic expression units (pDF124) were significantly lower compared to the two-promoter set-up (pDF141) (Fig. 2B) which made FACS-based quantitative analysis rather difficult (Figure 2C). However qualitative fluorescence microscopy suggested excellent regulation performance in individual transduced cells (Fig. 2B).

### Engineered AAV-derived particles transducing tightly regulated production of secreted proteins

**Figure 3:**
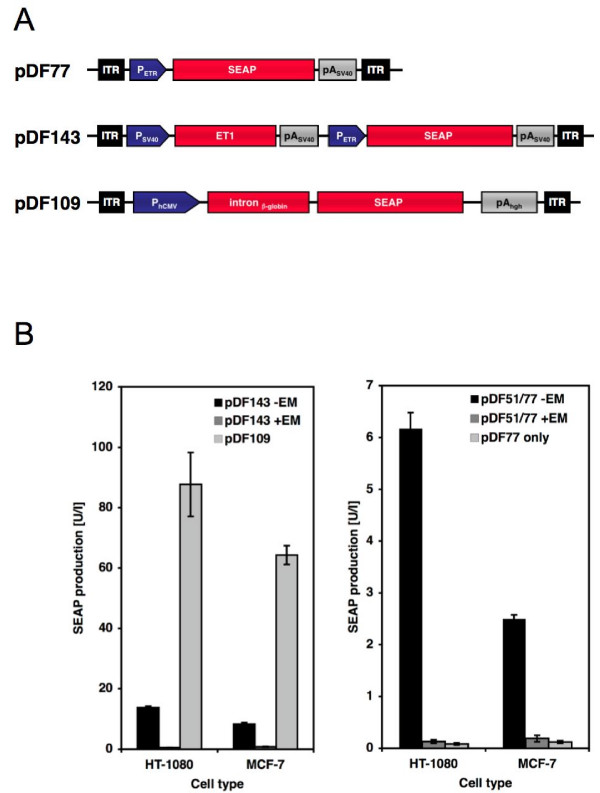
AAV type 2-based regulated expression of secreted proteins. (A) Schematic representation of pDF77, harboring a SEAP expression cassette under the control of P_ETR_, pDF143, a self-regulated expression unit expressing ET1 from P_SV40 _and driving SEAP from P_ETR _and pDF109, harboring a constitutive SEAP expression unit driven by P_hCMV _(B) SEAP expression levels profiled 48 h after (co-)transduction of transgenic AAV particles (1000 genomic particles/cell for pDF51/77, 1000 genomic particles/cell for pDF143 and 1000 genomic particles/cell for pDF109) derived from indicated vectors and cultivated in the presence (+) or absence (-) of EM. SEAP expression is shown in units/liter (U/l) as defined by Schlatter et al. [52]. Abbreviations: EM, erythromycin; ET1, erythromycin transactivator; ITR, inverted terminal repeat; pA_SV40_, simian virus 40 polyadenylation signal; P_hEF1α_, human elongation factor 1α promoter; P_ETR_, erythromycin-responsive promoter; SEAP, human placental secreted alkaline phosphatase; P_SV40_, simian virus 40 promoter.

Tight regulation of therapeutic transgenes remains a major challenge for current gene therapy initiatives. In order to assess whether AAV-based transduction systems enable delivery of tightly regulated expression of a human model glycoprotein, we constructed pDF77 harboring a P_ETR_-driven SEAP (human placental secreted alkaline phosphatase) (ITR-P_ETR_-SEAP-pA_SV40_-ITR) (Figure 3A). Co-transduction of HT-1080 and MCF-7 with pDF77- and pDF51- (ITR-P_hCMV_-ET1-pA_SV40_-ITR) derived AAV particles provided excellent regulation performance characterized by high-level SEAP production in the absence of erythromycin and repressed SEAP levels in the presence of erythromycin (Figure 3B). Furthermore, we have designed and evaluated pDF143 (ITR-P_SV40_-ET1-pA_SV40_-P_ETR_-SEAP-pA_SV40_-ITR), a one-vector expression configuration which is isogenic to pDF141 but encoding SEAP instead of EYFP. pDF143 exhibited similar SEAP regulation profiles in HT-1080 and MCF-7 compared to co-transduction of pDF77-/pDF51-derived AAV particles suggesting that there is no interference between ET1-driving P_SV40 _and ET1-specific P_ETR _(Figure 3A and 3B). Overall, maximum expression levels for the one-vector configuration were even higher than for the binary setting likely because for the binary system two different particles have to transduce a single cell which is a more rare event. Transduction of HT-1080 and MCF-7 with a constitutive control vector, pDF109 (ITR-P_hCMV_-intron_β-globin_-SEAP-pA_hgh_-ITR) showed increased SEAP production compared to fully induced pDF143-derived particles, which might be associated with either higher promoter strenght or the β-globin intron.

### AAVs engineered for E_ON_-controlled transgene expression

**Figure 4:**
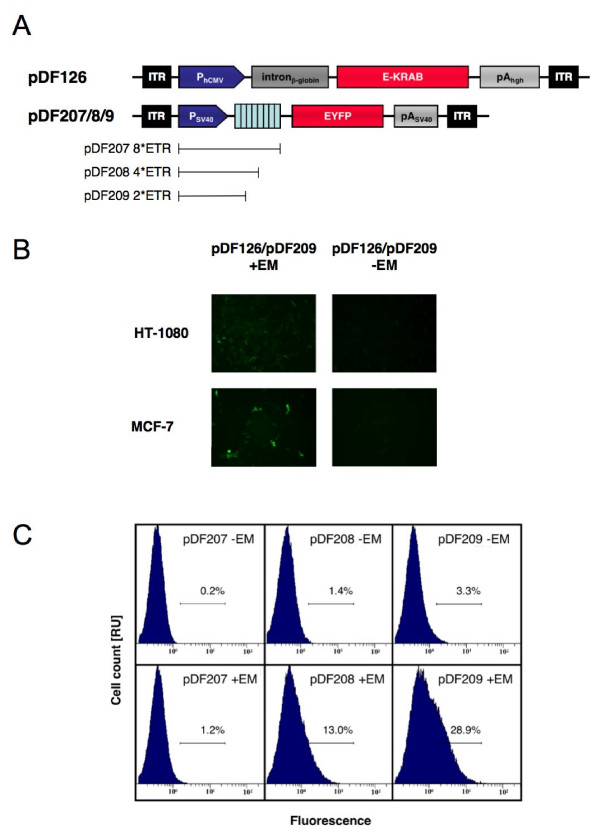
AAV type 2-based erythromycin-inducible EYFP expression. (A) Schematic representation of pDF126 harboring a P_hCMV_-driven E-KRAB expression unit and pDF206/207/208 encoding EYFP transcribed by P_ETR_ON promoter derivatives containing 8 (pDF207), 4 (pDF208) and 2 (pDF209) E-KRAB-specific ETR operator modules. (B) Fluorescence micrographs of HT-1080 and MCF-7 co-transduced with pDF209/pDF126-derived AAV particles (1000 genomic particles/cell for pDF207/208/209 and 4000 genomic particles/cell for pDF126) and cultivated in the presence (+EM) and absence (-EM) of erythromycin (C) FACS-mediated quantification of EYFP expression in HT-1080. EYFP-specific FACS diagrams of HT-1080 co-transduced (4000 and 1000 genomic particles/cell) by pDF126/pDF207, pDF126/pDF208 and pDF126/pDF209 were cultivated for 48 h in the presence (+) and absence (-) of EM. Abbreviations: EM, erythromycin; EYFP, enhanced yellow fluorescent protein; ITR, inverted terminal repeat; KRAB, kruppel associated box; P_ETR_ON, erythromycin-inducible promoter.

Various therapeutic initiatives require transgene control systems, which trigger heterologous target genes after administration of the regulating agent and remain repressed in the absence of the inducer [39]. We have constructed binary E_ON_-controlled AAV-based expression systems consisting of pDF126 (ITR-P_hCMV_-Intron-β-globin-E-KRAB-pA_hgh_-ITR), and pDF207 (ITR-P_ETR_ON8-EYFP-pA_SV40_-ITR), pDF208 (ITR-P_ETR_ON4-EYFP-pA_SV40_-ITR) or pDF209 (ITR-P_ETR_ON2-EYFP-pA_SV40_-ITR), which harbor EYFP under control of different E-KRAB-specific macrolide-inducible promoters characterized by different ETR tandem repeats placed 3' of the constitutive P_SV40 _promoter [14]. Functionality of the ON-type system was validated by co-transduction of pDF126/pDF209-derived into HT-1080 and MCF-7 and profiling of EYFP expression after 48 h cultivation in the presence or absence of erythromycin (Figure 4B). FACS-based comparison of HT-1080 co-transduced with pDF126-/pDF207-, pDF126-/pDF208- and pDF126-/pDF209-derived AAV particles and cultivated for 48 h in the presence and absence of erythromycin revealed that although overall induction factors were almost identical among different particle combinations, basal and maximum expression levels were a function of the number of ETR repeats associated with P_SV40 _(Figures 4A and 4C).

### Reversibility and adjustability of AAV-transduced macrolide-controlled transgene expression

Reversibility and adjustability are key characteristics for future clinical implementation of human-compatible transgene control modalities. In order to assess the reversibility of macrolide-responsive transgene expression in an AAV expression configuration we transduced HT-1080 and MCF-7 with pDF143-derived AAV particles and cultivated (i) in the presence (+++) or absence (---) of EM over 9 days, (ii) in the presence (++-) or absence (--+) of EM over 6 days and then incubated in reversed EM conditions for the remaining three days and (iii) in medium whose EM status was alternated every three days from +EM to -EM to +EM (+-+) or from -EM to +EM to -EM (-+-). SEAP accumulation was always measured prior to any EM status switch.

**Figure 5:**
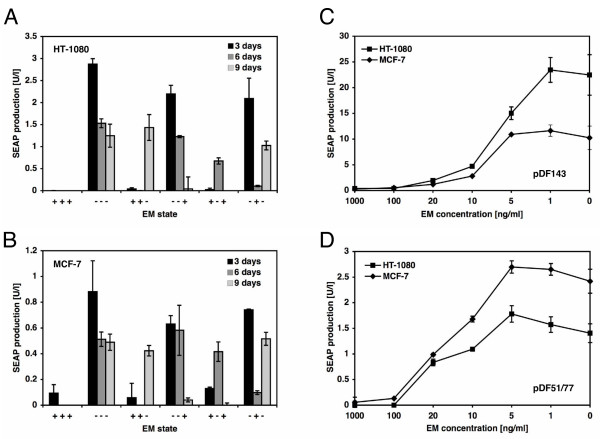
Reversibility and adjustability of macrolide-responsive SEAP expression in HT-1080 and MCF-7 transduced by transgenic AAV type 2-derived particles. (A) HT-1080 and (B) MCF-7 were transduced with pDF143-derived AAV particles (2000 genomic particles/cell) and six equal populations (1–6) were cultivated in media containing different antibiotic concentrations. Cells were (i) cultivated in the presence (+++) or absence (---) of EM over 9 days, (ii) cultivated in the presence (++-) or absence (--+) of EM over 6 days and then incubated in reversed EM conditions for the remaining three days or (iii) cells were cultivated in medium whose EM status was alternated every three days +EM to --EM to +EM (+-+) or from --EM to +EM to --EM (-+-) and SEAP expression was quantified. Adjustability of SEAP expression of pDF143 (1000 genomic particles/cell) (C) and pDF51/77 (500 genomic particles/cell) (D) in HT-1080 and MCF-7 cultivated for 48 h in the presence of increasing EM concentrations. SEAP expression is shown in units/liter (U/l) as defined by Schlatter et al. [52]. Abbreviations: EM, erythromycin; SEAP, human placental secreted alkaline phosphatase. Abbreviations: EM, erythromycin; SEAP, human placental secreted alkaline phosphatase.

HT-1080 and MCF-7 showed excellent switching characteristics upon application of removal of erythromycin (Figure 5A and 5B)

In order to assess the dose-response characteristics of binary and one-vector-based macrolide-responsive AAV transduction systems HT-1080 and MCF-7 were transduced with pDF143- or co-transduced with pDF77-/pDF51-derived AAV particles and cultivated for 48 h in the presence of different EM concentrations before SEAP production was quantified. Whereas SEAP expression was completely repressed between 20 and 100 ng/ml for both configurations gradual decrease in antibiotic treatment resulted in dose-dependent increase of transgene expression until maximum expression levels were reached in the absence of EM (Figure 5C and 5D). The differences in the dose-response characteristics of one-vector and binary configurations may result from different ET1-P_ETR _stoichiometries associated with those technologies.

### Transgenic AAV-derived particles mediate EOFF-controlled transgene expression in mice

**Figure 6:**
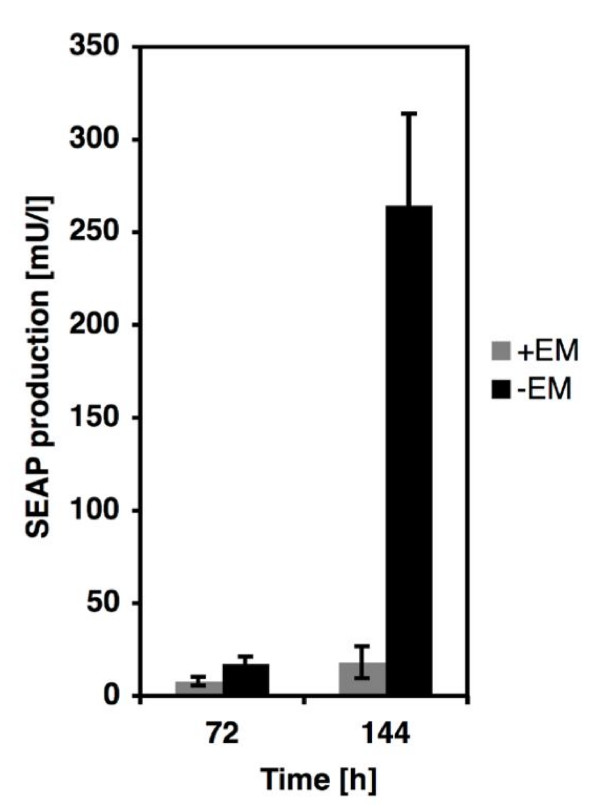
Macrolide-triggered SEAP expression in mice injected with pDF143 (ITR-P_SV40_-ET1-pA_SV40_-P_ETR_-SEAP-pA_SV40_-ITR)-derived AAV type 2 particles. pDF143-derived AAV particles were administered intramuscularly and SEAP levels in the serum were measured at two different time points for one group in the absence of erythromycin, and for the other group with EM injections every 24 h. SEAP expression is shown in milliunits/liter (mU/l) as defined by Schlatter et al. [52]. Abbreviations: EM, erythromycin; SEAP, human placental secreted alkaline phosphatase.

For *in-vivo *validation of AAV-based macrolide-responsive transgene transduction we injected 5*10^10 ^pDF143-derived genomic AAV particles intramuscularly into mice and treated them optionally with intraperitoneal erythromycin injections (2 mg/mouse every 24 h). Profiling of serum SEAP levels of treated mice showed significant production of this glycoprotein in mice, which had not received EM doses whereas SEAP production was repressed whenever EM was administered (Figure 6).

## Discussion

In recent years, gene delivery systems based on adeno-associated viral vectors have become one of the most promising tools for delivering transgenes *in vivo*. After major setbacks in trials with retroviral as well as adenoviral vectors [40,41], AAVs present a strong alternative for the safe and efficient delivery of transgenes. We have pioneered the delivery and regulation of transgenes by integrating the recently developed erythromycin-controllable E.REX system into AAV type 2-derived backbones. Considering that erythromycin is a clinically licensed drug and that AAV vectors have an excellent safety profile record, such a system is suitable for *in vivo *applications and is promising for clinical initiatives. Maximum erythromycin doses of up to 4 g/day in adults are in accordance with FDA and AHFS guidelines [42,43]. These doses are within the range of the amounts we have tested in mice. We are therefore confident that the AAV-encoded and erythromycin-controlled transgenes will be compatible with future clinical applications.

Research, in which AAV vectors deliver adjustable transgenes, has been conducted mainly with tetracycline responsive systems, which trigger or repress expression upon the addition of tetracycline [28,29,31,44,45].

We have designed a range of AAV type 2-based vectors encoding different configurations of the E_OFF _expression system to evaluate optimal arrangements for further *in vivo *studies. A binary set-up, where the transactivator is delivered on one vector and the gene of interest is driven by P_ETR _on another vector, worked well *in vitro*. The expression levels, vector titers and regulation performance of the tested configurations were excellent, indicating an efficient co-transduction of the two types of transgenic AAV particles. Regardless of whether intracellular or secreted transgenes were expressed, the regulation factors of all the tested cell lines were between 10 and 400. Although transgene expression mediated by AAVs engineered for macrolide-responsive transgenes expression were typically lower after induction compared to isogenic AAV derivatives containing constitutive promoters the overall production levels were in the same order of magnitude *in vitro*. Isogenic E_ON _systems also enabled excellent regulation performance but overall induction factors were typically lower, since cells exclusively transduced with the transgene-encoding AAV particle exhibit constitutive expression. Therefore, to achieve optimized E_ON_-controlled transgene expression from transgenic AAVs arranged in a binary vector set, the transrepressor-encoding AAV has to be administered in excess compared to the transgene-encoding counterpart or cell lines have to be used, which are particular susceptible to AAV-based transduction.

Generally, even though binary systems have the disadvantage that two vectors must enter the same cell to obtain regulated expression, this design is advantageous for some applications, namely when delivering large transgenes. Although binary systems are powerful tools, in a therapeutic setting it may be desirable to combine the regulation modules on a single vector. The advantages of such a setting are: (i) only one virus must enter the target cell to obtain regulated expression of the transgene, (ii) exact virus titration is easier and (iii) lower virus doses are required for comparable transgene expression.

We have pioneered both self-regulated and auto-regulated macrolide-responsive recombinant adeno-associated viral vectors for tightly controlled transgene expression. Although auto-regulated expression units are more compact than self-regulated configurations we had difficulty achieving reasonably high transduction efficiencies and expression levels. Since qRT-PCR-based analysis of viral particles revealed identical number of encapsidated genomes among our AAV portfolio, the lower transduction rates of auto-regulated AAVs may result from their genetic configuration rather than virus assembly. In contrast, self-regulated expression units, consisting of a strong constitutive promoter driving the expression of the transactivator as well as a macrolide-responsive promoter driving the desired transgene on the same vector, showed excellent performance in a variety of cell types. The vector design using compact promoter and polyadenylation elements in a self-regulated configuration allows the integration of a transgene with a size of up to 2500 bp which is sufficient for various clinically important transgenes (for example, erythropoietin or vascular endothelial growth factor). We did not observe promoter interference in the tested cell types, although the strong constitutive P_SV40 _promoter driving ET1 and the erythromycin-responsive promoter were on the same construct. We were successful in expressing intracellular as well as secreted transgenes in a highly controlled manner and showed precise titration of transgene expression upon addition of varying erythromycin concentrations as well as reversibility upon the addition or removal of the regulating agent. After switching from a fully repressed to a fully induced state or *vice versa *a short lag phase was observed before the new expression state was reached. This behaviour might be due to trace amounts of antibiotic remaining in the culture (by switching from +EM to -EM) or due to the degradation kinetics of transcripts and transactivators (from -EM to +EM)

One-vector configurations would be suitable for *in vivo *studies and would enable efficient targeting of a wide variety of cells by systemic administration or by local injection of the viral particles encoding the therapeutic transgene. We exemplified the functionality of the system *in vivo *by administrating pDF143-derived AAV particles intramuscularly to mice and analyzing transgene expression in the presence and absence of antibiotic for several days.

Most current studies on regulated transgene expression are carried out using retroviral vectors [46-50]. Although these systems are powerful and allow the efficient long-term expression of transgenes, they have several drawbacks, such as (i) the necessity of retroviral vectors for integration into transcriptionally active regions, which may interfere with desired transgene control, (ii) random integration, possibly triggering oncogene activation and (iii) silencing of the integrated transgene upon integration into the host chromosome. Adeno-associated viral vectors on the contrary, remain mainly episomal after entering the target cell and, therefore, do not have aforementioned drawbacks, thus providing a powerful alternative for upcoming gene-therapy studies.

## Conclusion

We have designed an array of novel AAV type 2-based expression vectors, which enable safe and efficient transduction of mammalian cells for macrolide-adjustable transgene expression. We have pioneered binary ON- and OFF-type systems as well as compact bidirectional, auto-regulated and self-regulated one vector expression configurations for regulation of intracellular as well as secreted proteins in mammalian cells. We have also engineered a compact self-regulated AAV which demonstrated efficient transduction, expression and regulation of a reporter gene in mice.

## Methods

### Plasmid construction

Table 1 lists all the plasmids used in this study as well as detailed information on their construction and the length of the genetic elements.

**Table 1:** Plasmids used and designed in this study

Plasmid	Description and Cloning Strategy	Reference or source
pAAV-MCS	AAV transfer vector	Stratagene
pAAV-lacZ	AAV vector expressing lacZ	Stratagene
pDG	Helper construct encoding AAV Rep/Cap as well as Adeno virus E2A, E4 and VA.	[38]
pCF18	Plasmid containing ECFP driven by a tetracycline responsive promoter and EYFP driven by a pristinamycin responsive promoter	[53]
pCF19	Plasmid containing SEAP cassette	[54]
pCF125	Plasmid expressing ECFP and ET1 from a bidirectional promoter	[21]
pSS134	Plasmid containing SEAP cassette	unpublished
pWW43	Plasmid expressing E-KRAB	[14]
pWW76	Plasmid containing tricistronic expression configuration driven by P_ETR_ON8	[14]
pWW78	pTRIDENT1-based tricistronic expression vector for macrolide-responsive auto-regulated expression of up to two desired transgenes.	[55]
pBP141	Vector expressing SEAP and ET1 under tetracycline-responsive promoter: P_hCMV*-1_-SEAP-IRES_PV_-ET-pA	unpublished
pMF123	Plasmid encoding tricistronic expression cassette driven by a constitutive SV40 promoter.	[56]
pMF351	Lentiviral vector encoding EYFP driven by a constitutive hCMV promoter	[51]
pDF37	AAV2 vector containing a tricistronic P_ETR _driven expression unit. The entire expression unit from pWW73 was excised using *Ssp*I/*Xba*I, polished by Klenow and cloned into pAAV-lacZ which was *Not*I digested and Klenow polished before, thus resulting in pDF37 (ITR-P_ETR_-IRES_PV_-IRES_EMCV_-pA_SV40_-ITR).	this work
pDF51	AAV2 vector containing a constitutive hCMV driven ET1 cassette. ET1 was excised from pWW078 using *Eco*RI/*Hind*III and cloned into the corresponding sites of pAAV-MCS, thus resulting in pDF51 (ITR-P_ETR_-Intron_β-globin_-ET1-pA_hgh_-ITR).	this work
pDF54	AAV2 vector encoding EYFP driven by the erythromycin responsive P_ETR _promoter. P_ETR _was excised from pDF55 using *Acc*I/*Nhe*I and cloned into the corresponding sites of pDF60, thus resulting in pDF54 (ITR-P_ETR_-EYFP-pA_SV40_-ITR).	this work
pDF55	AAV2 vector encoding divergent expression units for ECFP driven by P_ETR _and ET1 driven by a HSP70 minimal promoter. The entire expression cassette was excised from pCF125 using *Eco*RV/*Xba*I and cloned into the *Hinc*II/*Spe*I sites of pDF60, thus resulting in pDF55 (ITR-pA_I_-ECFP←P_ETR_-ETR-P_HSP70min_→ET1-pA_SV40_-ITR).	
pDF56	AAV2 vector encoding ET1 driven by a constitutive SV40 promoter. ET1 was excised from pWW78 using *Eco*RI/*Xba*I and cloned into the *Eco*RI/*Spe*I sites of pDF63, thus resulting in pDF56 (ITR-P_SV40_-ET1-pA_SV40_-ITR).	this work
pDF60	AAV2 vector encoding EYFP driven by a constitutive hCMV promoter. P_hCMV_-EYFP was excised from pMF351 using *Xba*I/*Pac*I and cloned into *Nhe*I/*Pac*I sites of pDF63, thus resulting in pDF60 (ITR-P_hCMV_-EYFP-pA_SV40_-ITR).	this work
pDF61	AAV2 vector encoding SEAP driven by an erythromycin responsive P_ETR _promoter. SEAP was excised from pCF019 using *Nhe*I/*Cla*I and cloned into the *Nhe*I/*Bst*BI sites of pDF54, thus resulting in pDF61 (ITR-P_ETR_-SEAP-pA_SV40_-ITR).	this work
pDF63	AAV2 vector containing an SV40 promoter followed by an IRES_PV _and a IRES_EMCV _element. P_SV40_-IRES_PV_-IRES_EMCV _was excised from pMF123 using *Ssp*I/*Bgl*II, polished with Klenow and cloned into the polished NcoI/SpeI sites of pAAV-lacZ, thus resulting in pDF63 (ITR- P_SV40_-IRES_PV_-IRES_EMCV_-pA_SV40_-ITR).	this work
pDF74	AAV2 vector containing tricistronic expression cassette driven by a P_ETR_ON8 promoter. The P_ETR_ON8 promoter was excised from pWW76 using *Nhe*I/*Eco*RI and cloned into the corresponding sites of pDF63, thus resulting in pDF74 (ITR-P_ETR_ON8-IRES_PV_-IRES_EMCV_-pA_SV40_-ITR).	this work
pDF75	AAV2 vector encoding dicistronic expression unit consisting of SEAP followed by an IRES_PV _element followed by ET1 driven by P_ETR_. Dicistronic expression cassette was excised from pBP141 using *Xba*I/*Pac*I and cloned into the *Nhe*I/*Pac*I sites of pDF54, thus resulting in pDF75 (ITR-P_ETR_-SEAP-IRES_PV_-ET1-pA_SV40_-ITR).	this work
pDF76	AAV2 vector encoding SEAP driven by a P_ETR_ON8 promoter. SEAP was excised from pSS134 using *Eco*RI/*Hind*III and cloned into pDF37. This vector was digested using *Eco*RI/*Asc*I and the SEAP containing insert was cloned into the corresponding sites of pDF74, thus resulting in pDF76 (ITR-P_ETR_ON8-SEAP-IRES_EMCV_-pA_SV40_-ITR).	this work
pDF77	AAV2 vector encoding SEAP under the control of an erythromycin responsive P_ETR _promoter (additional upstream ATG deleted). P_ETR _was excised from pDF54 using *Acc*I/*Eco*RI and cloned into the *Cla*I/*Eco*RI sites of pDF61, thus resulting in pDF77 (ITR-P_ETR_-SEAP-pA_SV40_-ITR)	this work
pDF89	AAV2 vector encoding divergent expression units for EYFP driven by P_ETR _and ET1 driven by a HSP70 minimal promoter. The EYFP cassette was excised from pCF18 using *Acc*I/*Eco*RV and cloned into the *Nru*I/*Cla*I sites of pDF55, thus resulting in pDF89 (ITR-pA_I_-EYFP←P_ETR_-ETR-P_HSP70min_→ET1-pA_SV40_-ITR)	this work
pDF98	Plasmid containing hEF1α promoter flanked by multiple cloning sites	unpublished
pDF109	AAV2 vector encoding SEAP driven by a constitutive hCMV promoter. SEAP was excised from pDF61 using *Eco*RI/*Spe*I and cloned into the *Eco*RI/*Xba*I sites of pAAV-MCS, thus resulting in pDF109 (ITR-P_hCMV_-Intron_β-globin_-SEAP-pA_hgh_-ITR)	
pDF124	AAV2 vector encoding dicistronic expression unit consisting of EYFP followed by an IRES_EMCV _element followed by ET1. The IRES-ET1 containing insert was excised from pDF75 using *Hind*III, polished with *Pfu *polymerase, digested using *Bst*XI and cloned into the *Swa*I/*Bst*XI sites of pDF54, thus resulting in pDF124 (ITR-P_ETR_-EYFP-IRES_EMCV_-ET1-pA_SV40_-ITR).	this work
pDF126	AAV2 vector encoding E-KRAB under the control of a constitutive hCMV promoter. The E-KRAB containing insert was excised from pWW043 using *Eco*RI/*Hpa*I and cloned into the *Eco*RI/*Hinc*II sites of pAAV-MCS, thus resulting in pDF126 (ITR-P_hCMV_-Intron_β-globin_-E-KRAB-pA_hgh_-ITR).	this work
pDF141	AAV2 vector encoding self-regulated expression cassette consisting of ET1 driven by a constitutive SV40 promoter and EYFP driven by P_ETR_. The entire ET1 expression cassette of pDF56 was excised using *Cla*I/*Pml*I and cloned into pDF54 which was digested by *Hind*III and polished by *Pfu *before, thus resulting in pDF141 (ITR-P_SV40_-ET1-pA_SV40_-P_ETR_-EYFP-pA_SV40_-ITR).	this work
pDF143	AAV2 vector encoding self-regulated expression cassette consisting of ET1 driven by a constitutive SV40 promoter and SEAP driven by P_ETR_. The SEAP containing insert was excised from pDF77 using *Kpn*I/*Spe*I and cloned into the corresponding sites of pDF141, thus resulting in pDF143 (ITR-P_SV40_-ET1-pA_SV40_-P_ETR_-SEAP-pA_SV40_-ITR).	this work
pDF199	AAV2 vector encoding SEAP under the control of a constitutive SV40 promoter followed by 2 binding sites for the transrepressor E-KRAB. The 4*ETR binding site containing fragment was excised from pWW55 using *Bst*BI/*Nde*I and cloned into the corresponding sites of pDF76. Two of the binding sites were deleted by recombination during the cloning procedure, thus resulting in pDF199 (ITR-P_ETR_ON2-SEAP-IRES_EMCV_-pA_SV40_-ITR).	this work
pDF200	AAV2 vector encoding SEAP under the control of a constitutive SV40 promoter followed by 4 binding sites for the transrepressor E-KRAB. The 4*ETR binding site-containing fragment was excised from pWW55 using *Bst*BI/*Nde*I and cloned into the corresponding sites of pDF76, thus resulting in pDF200 (ITR-P_ETR_ON4-SEAP-IRES_EMCV_-pA_SV40_-ITR).	this work
pDF207	AAV2 vector encoding EYFP under the control of a constitutive SV40 promoter followed by 8 binding sites for the transrepressor E-KRAB. EYFP was excised from pDF34 using *Eco*RI/*Pac*I and cloned into the corresponding sites of pDF76, thus resulting in pDF207 (ITR-P_ETR_ON8-EYFP-pA_SV40_-ITR).	this work
pDF208	AAV2 vector encoding EYFP under the control of a constitutive SV40 promoter followed by 4 binding sites for the transrepressor E-KRAB. EYFP was excised from pDF34 using *Eco*RI/*Pac*I and cloned into the corresponding sites of pDF200, thus resulting in pDF208 (ITR-P_ETR_ON4-EYFP-pA_SV40_-ITR).	this work
pDF209	AAV2 vector encoding EYFP under the control of a constitutive SV40 promoter followed by 2 binding sites for the transrepressor E-KRAB. EYFP was excised from pDF34 using *Eco*RI/*Pac*I and cloned into the corresponding sites of pDF199, thus resulting in pDF209 (ITR-P_ETR_ON2-EYFP-pA_SV40_-ITR).	this work

Abbreviations: **AAV2**, adeno-associated virus type 2; **ECFP**, enhanced cyan fluorescent protein (720 bp); **EM**, erythromycin; **ET1**, erythromycin transactivator (972 bp); **EYFP**, enhanced yellow fluorescent protein (720 bp); **IRES**_**EMCV**_, internal ribosome entry site of encephalomyocarditisviral origin (502 bp); **IRES**_**PV**_, internal ribosome entry site of polioviral origin (635 bp); **ITR**, inverted terminal repeat (141 bp); **KRAB**, kruppel-associated box (450 bp); **pA**_**I**_, artificial polyadenylation signal (91 bp); **pA**_**hgh**_, human growth hormone polyadenylation signal (478 bp); **pA**_**SV40**_, simian virus 40 polyadenylation signal (145 bp); **P**_**hEF1α**_, human elongation factor 1α promoter (1185 bp); **P**_**ETR**_, erythromycin responsive promoter (200 bp); **P**_**ETR**_**ON**, macrolide inducible promoter (530 bp); **P**_**hCMV**_, human immediate early cytomegalovirus promoter (663 bp); **P**_**HSP70min**_, heat shock protein 70 minimal promoter (350 bp); **P**_**SV40**_, simian virus 40 promoter (308 bp); **SEAP**, human placental secreted alkaline phosphatase (1560 bp)

### Cell culture and transfection

Human embryonic kidney cells, transgenic for the adenovirus type 5-derived E1 region as well as for the simian virus 40 (SV40) large T-antigen (HEK293-T; [51]), human fibrosarcoma cells (HT-1080; ATCC CCL-121), human breast cancer cells (MCF-7, ATCC HTB-22) and normal human dermal fibroblasts (NHDF; PromoCell, Heidelberg, Germany; cat. no. C-12300, lot no. 1070402) were cultivated in Dulbecco's modified Eagle's medium (DMEM, Invitrogen, Carlsbad, CA, USA) supplemented with 10% fetal calf serum (FCS; PAN Biotech GmbH, Aidenbach, Germany; cat. no. 3302-P231902, lot no. P231902) and 1% penicillin/streptomycin solution (Sigma Chemicals, St. Louis, MO, USA). Chinese hamster ovary cells (CHO-K1; ATCC CCL-61) were cultivated in ChoMaster^® ^HTS medium (Cell Culture Technologies GmbH, Gravesano, Switzerland) supplemented with 5% FCS (PAN Biotech GmbH) and 1% penicillin/streptomycin solution. All cell lines were cultivated at 37°C in a 5% CO_2_-containing humid atmosphere.

### Production of adeno-associated viral particles

AAV particles were produced by co-transfection of the helper plasmid pDG [38] and the transgene-encoding AAV vector into HEK293-T using optimized calcium phosphate transfection protocols. In brief, 2 × 10^6 ^HEK293-T were seeded per culture dish (diameter 10 cm) and cultivated overnight. For each plate 15 μg of helper plasmid were mixed with 5 μg AAV vector and CaCl_2 _was added to a final concentration of 0.25 M. The mixture was added slowly to an equal volume of HEPES-buffered saline solution (HeBS; 50 mM HEPES, 280 mM NaCl, 1.5 mM Na_2_HPO_4_, pH 7.1), vortexed briefly, and incubated for 2 min. before adding the DNA-calcium phosphate precipitate solution to the monolayer culture. Precipitates were removed after 6 h, cells were supplemented with 1% FCS-supplemented DMEM and incubated for 60 h. The supernatant was discarded, the cells were detached using a cell scraper and resuspended in 2 ml PBS (138 mM NaCl, 8.1 mM Na_2_HPO_4_, 1.47 mM KH_2_PO_4_, 2.67 mM KCl; Invitrogen, cat. no. 21600-069, lot. no. 1255481) per plate. Each cell suspension was transferred to a Falcon tube and pelleted by centrifugation for 3 min. at 280 × g. Following another washing step using 5 ml PBS, the cells were resuspended in 2 ml PBS and the intracellular viral particles were released after cell lysis induced by three consecutive freeze-thaw cycles consisting of shuttling the tubes between liquid nitrogen and a 37°C water bath (tubes were vortexed vigorously after each thawing step). The cell debris was eliminated by centrifugation for 5 min. at 10'000 × g and the supernatant containing the crude virus stock was collected, supplemented with 50 U/ml of Benzonase (Sigma, cat. no. E1014) and incubated for 30 min at 37°C. Iodixanol density-gradient purification of viral particles was performed using a protocol adapted from Zolotukhin et al. [37]. In brief, crude viral stocks were diluted in PBS to a final volume of 12 ml. Iodixanol step gradients were prepared in an ultracentrifuge tube by sequential underlaying of the crude viral preparation with 5 ml of a 15% (plus 1 M NaCl in the first layer), 5 ml of a 25%, 4 ml of a 40% and 4 ml of a 60% iodixanol-containing PBS. For better distinction of the gradient layers 2.5 μl/ml of a 0.5% Phenol Red (Sigma, cat. no. P0290) stock solution was added to the 60% and the 25% layers. Step gradients were centrifuged for 3.5 h at 150'000 × g at 18°C. The clear 40% fraction was harvested after puncturing the ultracentrifuge tube on the side with a syringe equipped with a 16G needle. A heparin affinity column (HiTrap Heparin HP, Amersham Biosciences, Sweden; cat. no. 17-0406-01) was equilibrated with 10 bed volumes of binding buffer (10 mM Na_2_PO_4_, pH 7) and run at a flow rate of 1 ml/min. The AAV particle-containing 40% fraction harvested from the step gradient was loaded onto the heparin affinity column. After washing the column with 10 bed volumes of binding buffer the viral particles were eluted with 5 bed volumes of elution buffer (10 mM Na_2_PO_4_, 1 M NaCl, pH 7) and then concentrated using a spin column with a molecular weight cut-off (MWCO) of 30 kDa (Vivaspin, Vivascience, Germany; cat. no. VS1521).

### Virus titration by quantitative real-time PCR

Crude viral preparations were treated as described in [36]. Primers and the Taqman probe were designed to anneal to (i) the SV40 polyadenylation signal (pA) (forward primer, 5'-AGCAATAGCATCACAAATTTCACAA-3', reverse primer, 5'-GACATGATAAGATACATTGATGAGTTTGG-3', Taqman FAM/TAMRA probe, 5'-AGCATTTTTTTCACTGCATTCTAGTTGTGGTTTG-3'), (ii) the SEAP open reading frame (forward primer, 5'-AGGCCCGGGACAGGAA-3', reverse primer, 5'-GCCGTCCTTGAGCACATAGC-3') or (iii) the erythromycin-dependent transactivator open reading frame (forward primer, 5'-CCAACTCCTCCAGGCACA-3', reverse primer, 5'-AGCAGGCCCTCGATGGTA-3'). Absolute quantification was performed using Taqman Universal PCR Master Mix (Applied Biosystems, Warrington, UK, cat. no. 4324018) or Power SYBR Green PCR Master Mix (Applied Biosystems, Warrington, UK, cat. no. 4367659) on AB Prism 7500 RT-PCR quantitative PCR hardware according to the manufacturer's instructions (Applied Biosystems, Weiterstadt, Germany). The reference standard consisted of a four log-spanning dilution of pDF60 harboring a single SV40 pA sequence, pDF51 harboring the ET1 transactivator or pDF109 harboring the SEAP open reading frame.

### Quantification of reporter gene expression

Enhanced yellow fluorescent protein (EYFP) was visualized using a Leica DM-RB fluorescence microscope (Leica Inc., Heerbrugg, Switzerland) equipped with an XF114 filter (Omega Optical Inc., Brattleboro, VT, USA). Fluorescence was quantified 48 h after transduction using a fluorescence-activated cell sorter (Coulter FC500, Beckman Coulter Inc., FL, USA) with CXP software (Beckman Coulter) installed. SEAP production was quantified in cell-culture supernatants 48 h after transduction and in mouse serum 3 days to 7 days after injection as described in [52].

### Chemicals used for transgene regulation

For all *in-vitro *experiments, erythromycin (Fluka, Buchs, Switzerland cat. no. E-5389) was prepared as a stock solution of 1 mg/ml in ethanol and used at a final concentration of 1 μg/ml. For *in-vivo *studies, 200 μl of a 10 mg/ml erythromycin solution (10% ethanol, 90% PBS) were daily intraperitoneally injected into each animal.

### *In vivo *studies

Female OF1 (oncins france souche 1) mice were obtained from Charles River Laboratories (Lyon, France). Mice were treated with intramuscular injections 5 × 10^10 ^vector genomes/mouse. Erythromycin was administered intraperitoneally 1 h after injection of the transgenic AAV particles and repeated every 24 h. Blood samples were collected retroorbitally and serum was produced using microtainer SST tubes (Beckton Dickinson, Plymouth, UK). All animal experiments were approved by the French Ministry of Agriculture and Fishery and performed by M.D.-E. at the Institut Universitaire de Technologie, IUTA, F-69622 Villeurbanne Cedex, France.

## Authors' contributions

DAF designed and performed all experiments except the animal studies which were conducted by MDE. MF participated in the conception and design of the study and helped to draft the manuscript. All authors read and approved the final manuscript.

## References

[B1] Edelstein ML, Abedi MR, Wixon J, Edelstein RM (2004). Gene therapy clinical trials worldwide 1989–2004-an overview. J Gene Med.

[B2] Tomanin R, Scarpa M (2004). Why do we need new gene therapy viral vectors? Characteristics, limitations and future perspectives of viral vector transduction. Curr Gene Ther.

[B3] Vandendriessche TC (2002). Clinical gene therapy – first international conference. 24–26 January 2002, Groningen, the Netherlands. IDrugs.

[B4] Li C, Bowles DE, van Dyke T, Samulski RJ (2005). Adeno-associated virus vectors: potential applications for cancer gene therapy. Cancer Gene Ther.

[B5] Grimm D, Kay MA (2003). From virus evolution to vector revolution: use of naturally occurring serotypes of adeno-associated virus (AAV) as novel vectors for human gene therapy. Curr Gene Ther.

[B6] Nakai H, Yant SR, Storm TA, Fuess S, Meuse L, Kay MA (2001). Extrachromosomal recombinant adeno-associated virus vector genomes are primarily responsible for stable liver transduction in vivo. J Virol.

[B7] Wang B, Li J, Xiao X (2000). Adeno-associated virus vector carrying human minidystrophin genes effectively ameliorates muscular dystrophy in mdx mouse model. Proc Natl Acad Sci USA.

[B8] Xu A, Song L, Wang C, Wang A, Xu Q, Xiao Z, Wang S, Li M, Hao S, Li Z (2000). [An observation of the immuno-persistence after inoculating with the domestic BRD II strain rubella vaccine among infants and young children]. Zhonghua Liu Xing Bing Xue Za Zhi.

[B9] Flotte TR, Solow R, Owens RA, Afione S, Zeitlin PL, Carter BJ (1992). Gene expression from adeno-associated virus vectors in airway epithelial cells. Am J Respir Cell Mol Biol.

[B10] Kaplitt MG, Leone P, Samulski RJ, Xiao X, Pfaff DW, O'Malley KL, During MJ (1994). Long-term gene expression and phenotypic correction using adeno-associated virus vectors in the mammalian brain. Nat Genet.

[B11] Gossen M, Bujard H (1992). Tight control of gene expression in mammalian cells by tetracycline-responsive promoters. Proc Natl Acad Sci USA.

[B12] Gossen M, Freundlieb S, Bender G, Muller G, Hillen W, Bujard H (1995). Transcriptional activation by tetracyclines in mammalian cells. Science.

[B13] Fussenegger M, Morris RP, Fux C, Rimann M, von Stockar B, Thompson CJ, Bailey JE (2000). Streptogramin-based gene regulation systems for mammalian cells. Nat Biotechnol.

[B14] Weber W, Fux C, Daoud-el Baba M, Keller B, Weber CC, Kramer BP, Heinzen C, Aubel D, Bailey JE, Fussenegger M (2002). Macrolide-based transgene control in mammalian cells and mice. Nat Biotechnol.

[B15] Zhao HF, Boyd J, Jolicoeur N, Shen SH (2003). A coumermycin/novobiocin-regulated gene expression system. Hum Gene Ther.

[B16] Rivera VM, Clackson T, Natesan S, Pollock R, Amara JF, Keenan T, Magari SR, Phillips T, Courage NL, Cerasoli F (1996). A humanized system for pharmacologic control of gene expression. Nat Med.

[B17] Braselmann S, Graninger P, Busslinger M (1993). A selective transcriptional induction system for mammalian cells based on Gal4-estrogen receptor fusion proteins. Proc Natl Acad Sci USA.

[B18] Beerli RR, Schopfer U, Dreier B, Barbas CF (2000). Chemically regulated zinc finger transcription factors. J Biol Chem.

[B19] Chartier C, Degryse E, Gantzer M, Dieterle A, Pavirani A, Mehtali M (1996). Efficient generation of recombinant adenovirus vectors by homologous recombination in Escherichia coli. J Virol.

[B20] Boorsma M, Nieba L, Koller D, Bachmann MF, Bailey JE, Renner WA (2000). A temperature-regulated replicon-based DNA expression system. Nat Biotechnol.

[B21] Fux C, Weber W, Daoud-El Baba M, Heinzen C, Aubel D, Fussenegger M (2003). Novel macrolide-adjustable bidirectional expression modules for coordinated expression of two different transgenes in mice. J Gene Med.

[B22] Weber W, Marty RR, Link N, Ehrbar M, Keller B, Weber CC, Zisch AH, Heinzen C, Djonov V, Fussenegger M (2003). Conditional human VEGF-mediated vascularization in chicken embryos using a novel temperature-inducible gene regulation (TIGR) system. Nucleic Acids Res.

[B23] Neddermann P, Gargioli C, Muraglia E, Sambucini S, Bonelli F, De Francesco R, Cortese R (2003). A novel, inducible, eukaryotic gene expression system based on the quorum-sensing transcription factor TraR. EMBO Rep.

[B24] Weber W, Rimann M, Spielmann M, Keller B, Daoud-El Baba M, Aubel D, Weber CC, Fussenegger M (2004). Gas-inducible transgene expression in mammalian cells and mice. Nat Biotechnol.

[B25] Acland GM, Aguirre GD, Bennett J, Aleman TS, Cideciyan AV, Bennicelli J, Dejneka NS, Pearce-Kelling SE, Maguire AM, Palczewski K (2005). Long-Term Restoration of Rod and Cone Vision by Single Dose rAAV-Mediated Gene Transfer to the Retina in a Canine Model of Childhood Blindness. Mol Ther.

[B26] Haberman RP, Samulski RJ, McCown TJ (2003). Attenuation of seizures and neuronal death by adeno-associated virus vector galanin expression and secretion. Nat Med.

[B27] Flotte T, Carter B, Conrad C, Guggino W, Reynolds T, Rosenstein B, Taylor G, Walden S, Wetzel R (1996). A phase I study of an adeno-associated virus-CFTR gene vector in adult CF patients with mild lung disease. Hum Gene Ther.

[B28] Chtarto A, Bender HU, Hanemann CO, Kemp T, Lehtonen E, Levivier M, Brotchi J, Velu T, Tenenbaum L (2003). Tetracycline-inducible transgene expression mediated by a single AAV vector. Gene Ther.

[B29] Gafni Y, Pelled G, Zilberman Y, Turgeman G, Apparailly F, Yotvat H, Galun E, Gazit Z, Jorgensen C, Gazit D (2004). Gene therapy platform for bone regeneration using an exogenously regulated, AAV-2-based gene expression system. Mol Ther.

[B30] Bohl D, Salvetti A, Moullier P, Heard JM (1998). Control of erythropoietin delivery by doxycycline in mice after intramuscular injection of adeno-associated vector. Blood.

[B31] Jiang L, Rampalli S, George D, Press C, Bremer EG, O'Gorman MR, Bohn MC (2004). Tight regulation from a single tet-off rAAV vector as demonstrated by flow cytometry and quantitative, real-time PCR. Gene Ther.

[B32] Rivera VM, Gao GP, Grant RL, Schnell MA, Zoltick PW, Rozamus LW, Clackson T, Wilson JM (2005). Long-term pharmacologically regulated expression of erythropoietin in primates following AAV-mediated gene transfer. Blood.

[B33] Wang J, Voutetakis A, Papa M, Rivera VM, Clackson T, Lodde BM, Mineshiba F, Baum BJ (2005). Rapamycin control of transgene expression from a single AAV vector in mouse salivary glands. Gene Ther.

[B34] Grimm D, Kern A, Rittner K, Kleinschmidt JA (1998). Novel tools for production and purification of recombinant adenoassociated virus vectors. Hum Gene Ther.

[B35] Xiao X, Li J, Samulski RJ (1998). Production of high-titer recombinant adeno-associated virus vectors in the absence of helper adenovirus. J Virol.

[B36] Clark KR, Liu X, McGrath JP, Johnson PR (1999). Highly purified recombinant adeno-associated virus vectors are biologically active and free of detectable helper and wild-type viruses. Hum Gene Ther.

[B37] Zolotukhin S, Byrne BJ, Mason E, Zolotukhin I, Potter M, Chesnut K, Summerford C, Samulski RJ, Muzyczka N (1999). Recombinant adeno-associated virus purification using novel methods improves infectious titer and yield. Gene Ther.

[B38] Grimm D, Kay MA, Kleinschmidt JA (2003). Helper virus-free, optically controllable, and two-plasmid-based production of adeno-associated virus vectors of serotypes 1 to 6. Mol Ther.

[B39] Weber W, Fussenegger M (2006). Pharmacologic transgene control systems for gene therapy. J Gene Med.

[B40] Check E (2002). Regulators split on gene therapy as patient shows signs of cancer. Nature.

[B41] Lehrman S (1999). Virus treatment questioned after gene therapy death. Nature.

[B42] Food and Drug Administration. http://www.fda.gov/.

[B43] AHF Drug Information. http://www.ashp.org/ahfs/index.cfm.

[B44] Stieger K, Le Meur G, Lasne F, Weber M, Deschamps JY, Nivard D, Mendes-Madeira A, Provost N, Martin L, Moullier P (2006). Long-term doxycycline-regulated transgene expression in the retina of nonhuman primates following subretinal injection of recombinant AAV vectors. Mol Ther.

[B45] Haberman RP, McCown TJ, Samulski RJ (1998). Inducible long-term gene expression in brain with adeno-associated virus gene transfer. Gene Ther.

[B46] Markusic D, Oude-Elferink R, Das AT, Berkhout B, Seppen J (2005). Comparison of single regulated lentiviral vectors with rtTA expression driven by an autoregulatory loop or a constitutive promoter. Nucleic Acids Res.

[B47] Mitta B, Weber CC, Fussenegger M (2005). In vivo transduction of HIV-1-derived lentiviral particles engineered for macrolide-adjustable transgene expression. J Gene Med.

[B48] Mitta B, Weber CC, Rimann M, Fussenegger M (2004). Design and in vivo characterization of self-inactivating human and non-human lentiviral expression vectors engineered for streptogramin-adjustable transgene expression. Nucleic Acids Res.

[B49] Pluta K, Luce MJ, Bao L, Agha-Mohammadi S, Reiser J (2005). Tight control of transgene expression by lentivirus vectors containing second-generation tetracycline-responsive promoters. J Gene Med.

[B50] Vigna E, Amendola M, Benedicenti F, Simmons AD, Follenzi A, Naldini L (2005). Efficient Tet-dependent expression of human factor IX in vivo by a new self-regulating lentiviral vector. Mol Ther.

[B51] Mitta B, Rimann M, Ehrengruber MU, Ehrbar M, Djonov V, Kelm J, Fussenegger M (2002). Advanced modular self-inactivating lentiviral expression vectors for multigene interventions in mammalian cells and in vivo transduction. Nucleic Acids Res.

[B52] Schlatter S, Rimann M, Kelm J, Fussenegger M (2002). SAMY, a novel mammalian reporter gene derived from Bacillus stearothermophilus alpha-amylase. Gene.

[B53] Fux C, Fussenegger M (2003). Toward higher order control modalities in mammalian cells-independent adjustment of two different gene activities. Biotechnol Prog.

[B54] Fux C, Langer D, Fussenegger M (2004). Dual-regulated myoD- and msx1-based interventions in C2C12-derived cells enable precise myogenic/osteogenic/adipogenic lineage control. J Gene Med.

[B55] Weber W, Marty RR, Keller B, Rimann M, Kramer BP, Fussenegger M (2002). Versatile macrolide-responsive mammalian expression vectors for multiregulated multigene metabolic engineering. Biotechnol Bioeng.

[B56] Fussenegger M, Mazur X, Bailey JE (1998). pTRIDENT, a novel vector family for tricistronic gene expression in mammalian cells. Biotechnol Bioeng.

